# Two new species of *Amphinemura* (Plecoptera, Nemouridae) from the southern Qinling Mountains of China, based on male, female and larvae

**DOI:** 10.3897/zookeys.808.29433

**Published:** 2018-12-18

**Authors:** Weihai Li, Raorao Mo, Wenbin Dong, Ding Yang, Dávid urányi

**Affiliations:** 1 Department of Plant Protection, Henan Institute of Science and Technology, Xinxiang, Henan 453003, China Henan Institute of Science and Technology Henan China; 2 Guangxi key laboratory of Agric-Environment and Agric-Products Safety, Agricultural College, Guangxi University, Nanning, China Guangxi University Nanning China; 3 Department of Entomology, China Agricultural University, 2 Yuanmingyuan West Road, Beijing 100193, China China Agricultural University Beijing China; 4 Plant Protection Institute, Centre for Agricultural Research, Hungarian Academy of Sciences, Herman Ottó út 15, Budapest H-1022, Hungary Centre for Agricultural Research, Hungarian Academy of Sciences Budapest Hungary; 5 Department of Zoology, Hungarian Natural History Museum, Baross u. 13, Budapest H-1088, Hungary Hungarian Natural History Museum Budapest Hungary

**Keywords:** *
Amphinemura albicauda
*, *
A. dingoidea
*, Amphinemurinae, new records, new species, Shaanxi, Stoneflies

## Abstract

Two new species of the genus *Amphinemura*, *A.albicauda***sp. n.** and *A.dingoidea***sp. n.** from the southern Qinling Mountains, Foping County of Shaanxi Province, western China, are described based on both sexes and the larval stage. The new species are compared with related taxa, and the *A.sinensis* species group is defined for an Oriental lineage of the genus. *Amphinemurasinensis* (Wu, 1926) and *A.unihamata* (Wu, 1973) are reported from Shaanxi for the first time, and the hitherto unknown female of *A.unihamata* is described. A distribution map of the Amphinemurinae known from Qinling Mountains is given.

## Introduction

The subfamily Amphinemurinae belongs to the stonefly family Nemouridae. It is the second largest group in China (DeWalt et al. 2018, Yang and Li 2018, Yang et al. 2015). The most species rich genus, *Amphinemura* Ris, 1902, includes 85 described Chinese species (DeWalt et al. 2018). The monograph by Yang et al. (2015) treats all the species described by Klapálek (1912), Wu (1926, 1935, 1938, 1949, 1962, 1973), Martynov (1928), Shimizu (1997, 1998), Zhu and Yang (2002, 2003), Yang et al. (2004, 2005), Li and Yang (2005, 2006, 2007a, 2008a, b, c, d, e, 2011), Li et al. (2005), Wang et al. (2006, 2007), Du and Wang (2007), and Du et al. (2007b). Since then, Li et al. (2013, 2016b, 2017a, b, 2018), Ji and Du (2014), Ji et al. (2014), and Mo et al. (2017) have described an additional 17 species of *Amphinemura* from China.

Recent research has highlighted the high diversity of stonefly fauna of the poorly investigated Qinling Mountains, which range from the Qinghai-Tibet Plateau to the North China Plain and separate northern and southern China (Ji et al. 2014; Li and Murányi 2015; Li et al. 2016a, 2018; Li and Mo 2018). In April 2018, we made a short collecting trip to the southern Qinling Mountains, in the vicinity of Foping, Shaanxi. In the present paper, we describe two new species of *Amphinemura* collected during that trip, along with some further contributions to the Amphinemurinae, including the definition of a new Oriental species group where one of the new species belongs. We also present a distribution map of the Amphinemurinae hitherto known from the Qinling Mountains.

## Material and methods

The material studied was collected along stream banks by hand, or by using a beating sheet or an aquatic net. All material is deposited in the Entomological Museum of China Agricultural University, Beijing (CAUC), the Henan Institute of Science and Technology, Xinxiang (HIST), and the Collection of Smaller Insect Orders, Department of Zoology, Hungarian Natural History Museum, Budapest (HNHM), as indicated in the text. The specimens are preserved in 75% ethanol. The morphological terminology follows that of Baumann (1975) and Zwick (2010). Specimens were examined with the aid of a Leica M420 dissecting microscope and the color images and illustrations were made with the aid of a Leica S8APO and a Keyence LHX5000 digital microscope.

## Results and discussion

### Amphinemura albicauda sp. n.

Taxon classificationAnimaliaPlecopteraNemouridae

http://zoobank.org/2F1C3751-1EE0-4237-9B4D-7FB10A427220

Figs 1 , 2–6 , 7 , 8–13 , 14 , 31 , 32 , 35

#### Diagnosis.

Male: tergum IX with short spines and long setae, epiproct with closely spaced lateral processes having sharp and out-curved apex, paraproctal outer lobe long and armed with large apical teeth, median lobe apically bilobed. Female: subgenital plate subquadrate and slightly lobed, inner sclerite with ear-shaped lobes. Larva: general color brown but cerci contrasting white and hairy, legs with distinct swimming hairs.

#### Description.

**Figure 1.**
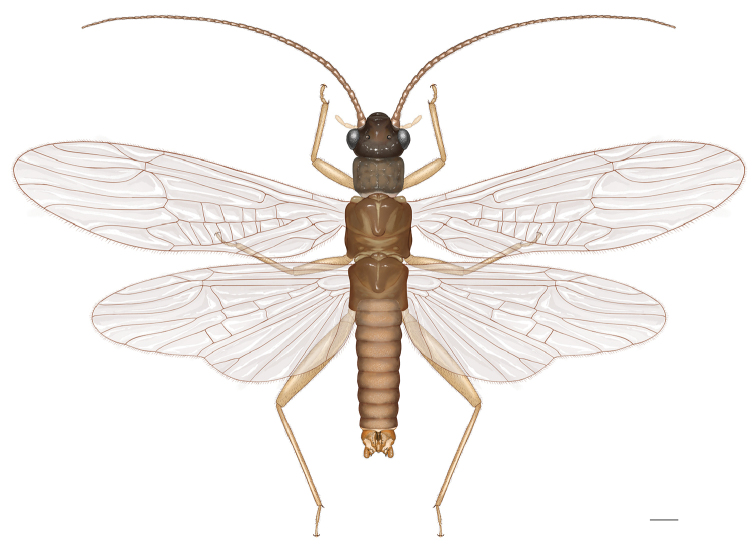
*Amphinemuraalbicauda* sp. n., habitus of male holotype adult. Scale bar: 0.5 mm.

Adult habitus (Fig. 1): General color light brown to brown. Head and antennae brown, palpi light brown. Thorax brownish, pronotum with distinct rugosities. Legs light brown. Wing membranes grayish, veins brown. Abdomen brown with darker terminalia.

**Figures 2–6.**
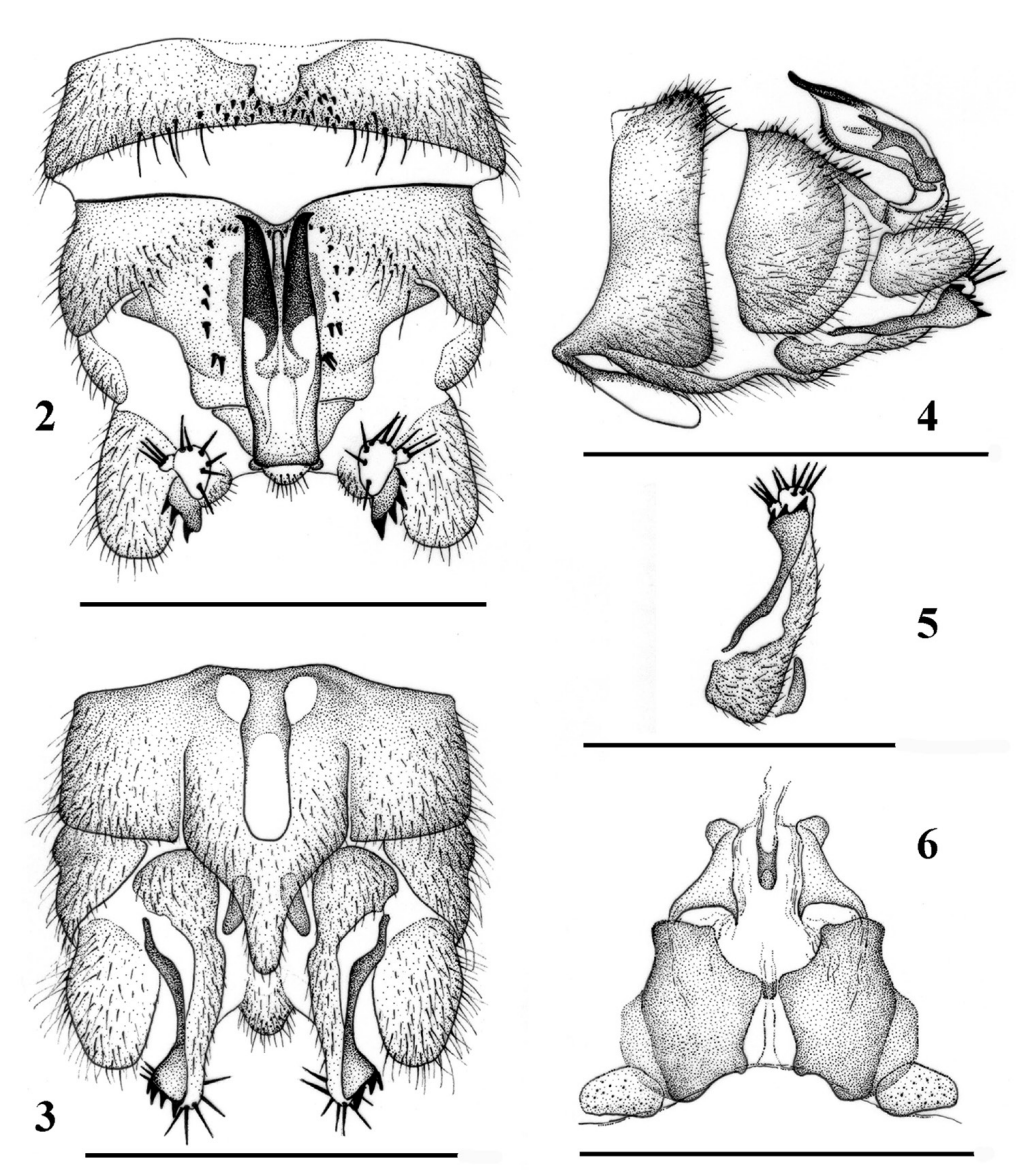
*Amphinemuraalbicauda* sp. n., terminalia of the adult paratypes. **2** Male terminalia, dorsal view **3** Male terminalia, ventral view **4** Male terminalia, lateral view **5** Male paraproct, oblique lateral view **6** Female inner genitalia, dorsal view. Scale bar: 0.5 mm.

Male (Fig. 2–5): Forewing length 6.4–6.6 mm. Tergum IX (Fig. 2) moderately sclerotized, with 11–13 short mesal spines and 4 or 5 paramedial long hairs along mid-posterior margin. Sternum IX with claviform vesicle, distal ½ membranous (Fig. 3). Hypoproct subquadrate at base, apical ½ tapering, apex tubular (Fig. 3). Tergum X weakly sclerotized, concavity beneath epiproct narrow with 10–12 small to medium-sized spines along lateral sides (Figs 1, 2). Cercus slightly sclerotized, stout and short. Epiproct (Figs 1, 2, 4) ca 3.5 times longer than wide, distal portion trifurcate but median process nearly unpigmented and hardly detected in dorsal view (Fig. 2). The lateral processes horn-shaped and closely located, with apex sharp and out-curved; median process originates from ventral sclerite and shorter than lateral ones, subapically forming distinct rounded ridge in lateral view (Fig. 4). Paraproct trilobed (Figs 2–5): inner lobe triangular, short and well sclerotized, mostly hidden by hypoproct; median lobe long and tubular, apical portion up-curved, apex membranous with 6 or 7 long marginal spines, outer margin with an additional small lobe bearing 3 long spines of same size; outer lobe extends along with median lobe but shorter, well sclerotized, dilated apex with 3 or 4 lateral, large teeth along outer margin.

Female (Fig. 14): Forewing length 7.8 mm. Sternum VII produced in a broad semicircular pregenital plate covering anterior half of subgenital plate; the plate is mostly pale with brownish posterior margin. Sternum VIII forms sclerotized subquadrate subgenital plate with narrowed anterior portion, medial notch is being broad but shallow medially; inner portion of the posterior lobes with small, sometimes indistinct secondary lobes. Paragenital plate paired, forming quadrate brownish lobe connected with posterolateral corner of subgenital plate. Sternum IX trapezoidal, median half much protruded anteriorly, in ventral aspect with anterior indentation. Paraproct and cerci brownish.

Female inner genitalia (Fig. 6): Inner sclerite is medially separated into triangular sclerites located anteriorly to the subgenital plate; anterior portion of the sclerite halves with a small, ear-shaped projection, easily observed in oblique or caudal view. Between the sclerites is a membranous tunnel with a tubular median sclerite leading to spermathecal ductus.

**Figure 7.**
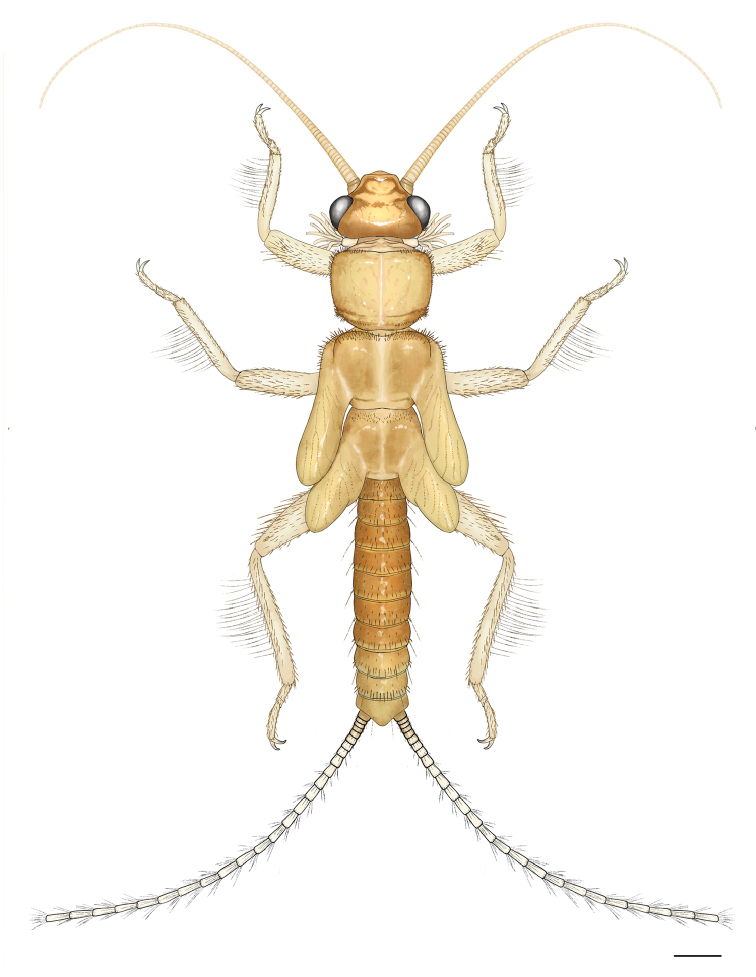
*Amphinemuraalbicauda* sp. n., habitus of matured female paratype larva. Scale bar: 0.5 mm.

Mature larva (Fig. 7): Body relatively slender and small, body length without antennae and cerci 5.5–6.0 mm. General color brown, with some indistinct pattern on pronotum and abdomen, legs and antennae light brown but cerci contrasting white and hairy. Setation long and distinct. Legs moderately long, width of hind femora ca ¼ of their length. The pronotum is trapezoidal, wider than long, as wide as head. Cervical gills long, inner gills with 7, outer with 8 branches. Wing pads more than twice as long as the corresponding segments. Abdomen relatively slender and uniformly brown, integument light matt brown, first 2 abdominal segments divided by pleura. Posterior margin of sternum IX of the male larva short triangular, sternum VIII of female larva slightly incised; paraprocts blunt. Cerci long, with 24–26 cylindrical segments; length of the 15^th^ segment is about 3 times of its width.

**Figures 8–13.**
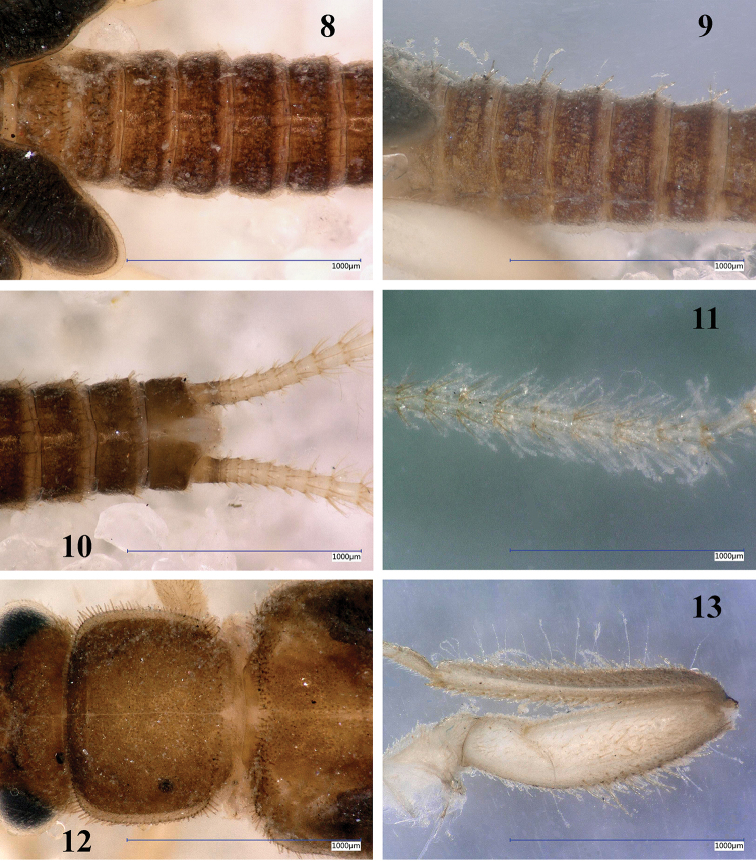
*Amphinemuraalbicauda* sp. n., matured larva paratypes. **8** Terga I–VI, dorsal view **9** Terga II–VII, lateral view **10** Terga VII–X and basal third of cerci, dorsal view **11** Cercomeres 9–18, dorsal view **12** Pronotum, dorsal view **13** Right hind leg, outer face. Scale bar: 1 mm.

Setation of the larva (Figs 8–13): Head, antennae and palpi with dense short setae. Pronotum covered with very short setae; marginal setae distinct and blunt, row interrupted in anteromedial and posteromedial third, corners have setae as long as one 15^th^ of pronotum width (Fig. 12). Setae on meso- and metanotum as long as longest marginal setae on pronotum; wing pads with long and acute, whitish setae. Legs with dense setation, all tibiae bear distinct swimming hairs as long as femur width (Fig. 13). Longest acute setae of all femora are longer than half of the corresponding femur width, long setae on fore femur arranged in an incomplete transversal line. Tarsi and claws relatively long. Tergal segments covered with thin setae, all segments bear a pair of thin, erect and irregularly curved hairs that are distinct in lateral view and reach nearly the segment length (Fig. 9); posterior margin with row of 14–16 acute setae, of various length, longest nearly reaches half of segment length (Figs 8, 10). Cercal segments with dense and long, white setation; setae sparser and shorter on basal and apical segments (Figs 10, 11). Cercomeres 14–16 with intercalary setae-like fine hairs, longer than the segment width, and an apical whorl of 14–17 acute setae that are as long as segment length (Fig. 11).

#### Type material.

Holotype male (HIST): CHINA, Shaanxi Province, Hanzhong, Foping County, Changjiaoba Town, lower section of a large unnamed stream in Dizhuang valley, 895 m, 33°33.344'N, 107°59.018'E, 2018.IV.21, leg. W.H. Li, R.R. Mo and D. Murányi. Paratypes: same data as holotype: 1 male and 1 female, 1 female larva (HIST), 1 male larva (CAUC) 1 male and 1 female larva, with pharate adult terminalia dissected (HNHM).

#### Affinities.

The new species belongs to a lineage of *Amphinemura* that is distributed in Oriental areas of China and Vietnam. This lineage can be characterized by the horn-shaped lateral processes of the dorsal sclerite, and pointed ventral sclerite of the male epiproct. It is defined here as the *A.sinensis* species group. The species belonging to this group are *A.ancistroidea* Li & Yang, 2007a, *A.caoae* Stark & Sivec, 2010, *A.cestroidea* Li & Yang, 2005, *A.chui* Wu, 1935, *A.divergens* Stark & Sivec, 2010, *A.elongata* Li, Yang & Sivec, 2005, *A.fleurdelia* (Wu, 1949), *A.furcostyla* (Wu, 1973), *A.giay* Stark & Sivec, 2010, *A.guangdongensis* Yang, Li & Zhu, 2004, *A.hamiornata* Li & Yang, 2008b, *A.leigong* Wang & Du in Wang et al. 2006, *A.licenti* (Wu, 1938), *A.malleicapitata* Li & Yang, 2006, *A.maoi* (Wu, 1938), *A.nanlingensis* Yang, Li & Sivec, 2005, *A.nigritubulata* Li & Yang, 2008d, *A.sinensis* (Wu, 1926), *A.tianmushana* Li & Yang, 2011, *A.viet* Stark & Sivec, 2010, and *A.yao* Mo, Yang, Wang & Li, 2017. Males of the new species can be distinguished from other members of the group by their armed outer lobe of the paraproct, which is unique within the group, and also by the distinctive shape of the processes of epiproct. The female outer genital sclerites are less distinctive, but the inner sclerite is unique because of the ear-shaped projections. The larva is distinctive by its conspicuous white and rather hairy cerci.

#### Distribution and ecology.

The new species was found at a single locality at the lower section of a large stream at moderate elevation (Figs 31, 35). The stream runs between forests, less-used agricultural areas, and ruderal bush. Its width varies between 3 and 8 m, and its maximum depth is less than 1 m. Rocky rapids are mixed with nearly stagnant pools and slow, stony sections. The substrate is mostly stony or sandy, with a moderate amount of debris (Fig. 32). Both last instar larvae and fully mature adults were present, suggesting April is the peak season of its emergence. Accompanying stoneflies were *A.sinensis*, a *Rhopalopsole* sp. collected as females, a few larvae of a *Neoperla* sp., and larvae of a *Kamimuria* sp. that occurred in high numbers.

#### Etymology.

The specific name is composed of the Latin words *albus* (white) and *cauda* (tail), and refers to the distinctive white cerci of the larva.

#### Remarks.

The adults and larvae were associated on the basis of pharate male and female adult terminalia dissected from matured larvae.

### Amphinemura dingoidea sp. n.

Taxon classificationAnimaliaPlecopteraNemouridae

http://zoobank.org/6C899234-61F5-4469-BAA6-2BED14A6EBAC

Figs 15 , 16 , 17–21 , 22 , 23–28 , 33–34 , 35

#### Diagnosis.

Male: tergum IX with long setae, ventral vesicle very long, epiproct weakly modified but with apical notch, paraproctal inner lobe long and slightly bilobed, median lobe long, curved and with 5 or 6 apical spines, outer lobe short and lacks spine. Female: subgenital plate strongly bilobed with dome-like median notch, paragenital plate with two branches, inner genitalia simple. Larva: general color light brown with specific, distinct dark brown dorsal pattern, setation long.

#### Description.

Adult habitus (Fig. 16): Head dark brown without pattern; compound eyes dark brown; antennae dark brown; palpi light brown. Pronotum lighter than head, arrangement of brown pattern similar to larvae. Legs generally brown, coxae and venter of femora lighter. Abdominal segments reddish brown, terminalia brown.

**Figures 14, 15.**
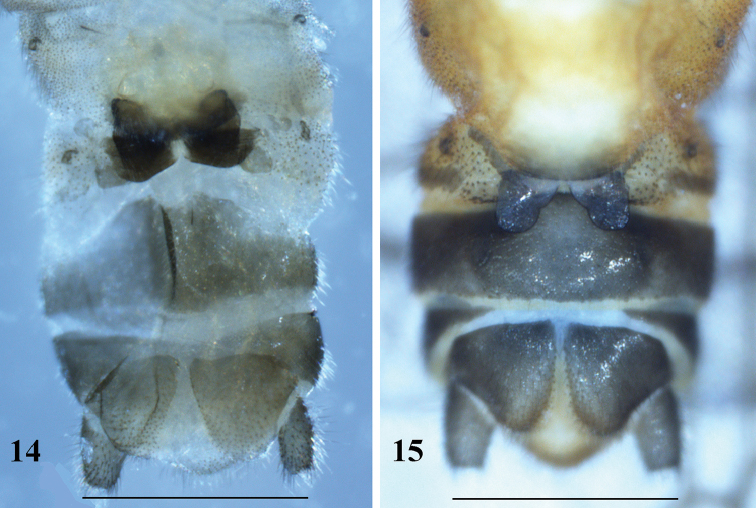
Female terminalia in ventral view **14***Amphinemuraalbicauda* sp. n., paratype **15***Amphinemuradingoidea* sp. n., paratype. Scale bar: 0.5 mm.

**Figure 16.**
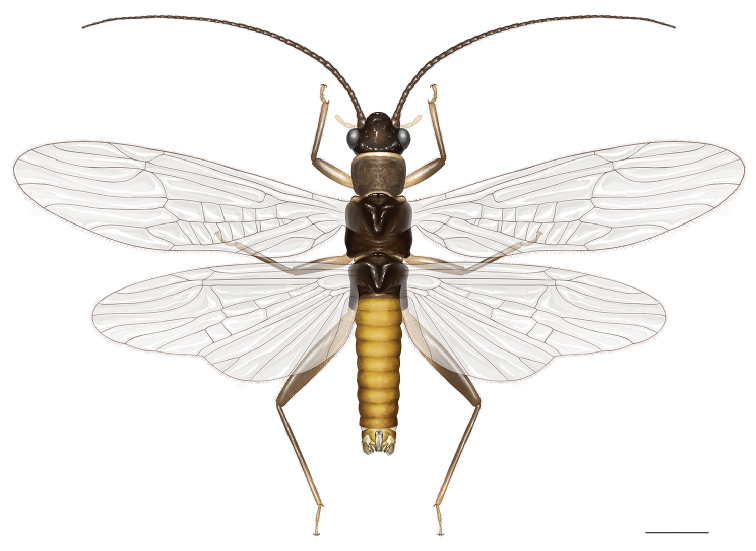
*Amphinemuradingoidea* sp. n., habitus of male holotype adult. Scale bar: 1 mm.

**Figures 17–21.**
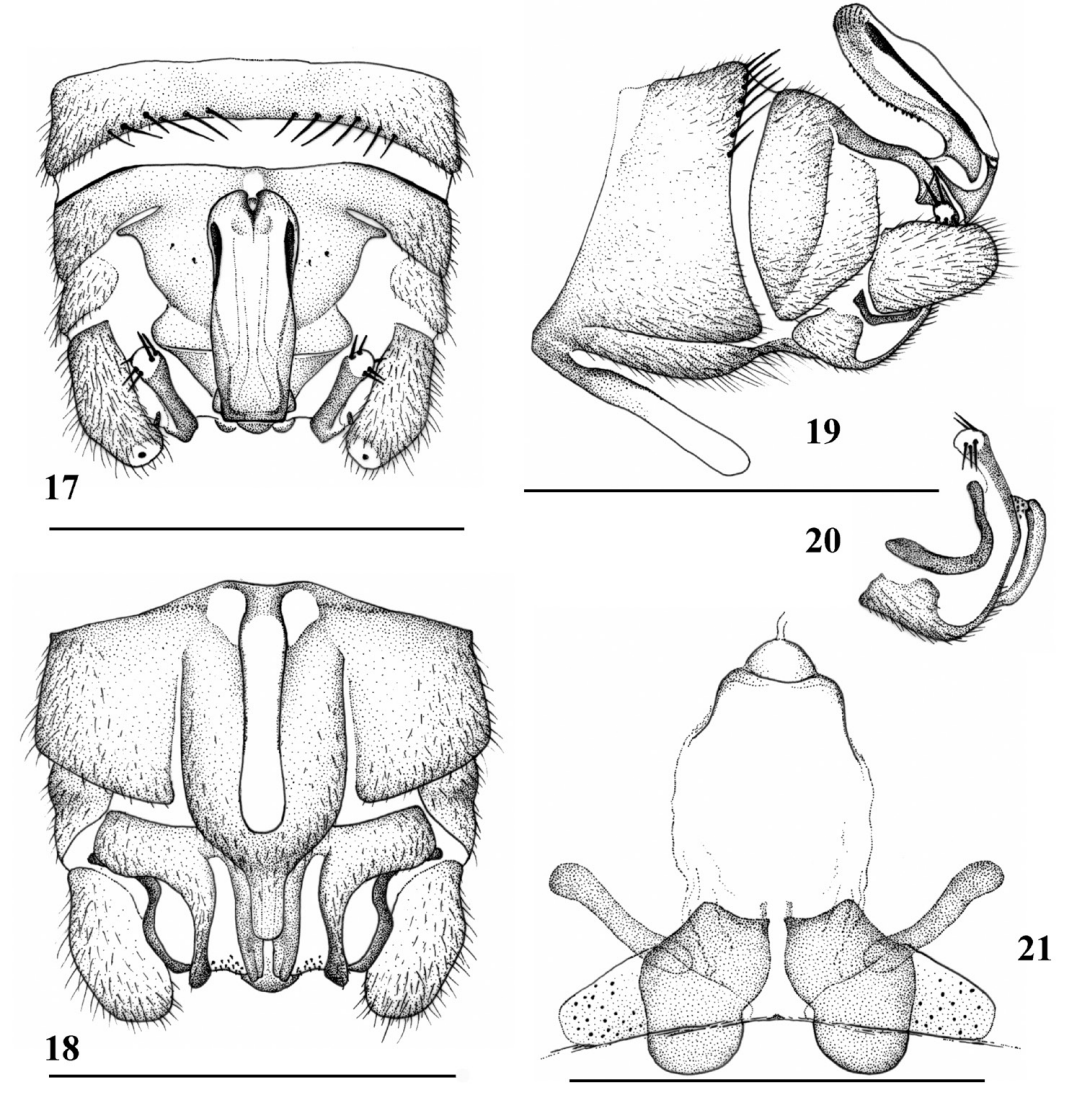
*Amphinemuradingoidea* sp. n., terminalia of the adult paratypes. **17** Male terminalia, dorsal view **18** Male terminalia, ventral view **19** Male terminalia, lateral view **20** Male paraproct, oblique lateral view **21** Female inner genitalia, dorsal view. Scale bar: 0.5 mm.

Male (Figs 17–20): Forewing length 6.3–6.5 mm. Tergum IX lightly but evenly sclerotized, two paramedial groups of 6–8 long setae present along posterior margin (Fig. 17). Vesicle of sternum IX very long, constricted medially, about 5 times longer than wide (Figs 18, 19). Hypoproct basal half rectangular, medial portion rounded, apex short and up-curved (Fig. 18). Tergum X widely sclerotized, medial light area beneath epiproct very small and narrow, with 3 or 4 very small lateral spinules (Fig. 17). Cercus lightly sclerotized, stout and short. Epiproct (Figs 16, 17, 19) weakly modified, nearly rectangular with lightly sinuous margin, less than 3 times longer than wide, apically scaled and with a dark medioapical notch. Dorsal sclerite mostly membranous, basal sclerite narrow, lateral sclerite evenly thin and S-curved, basal portion hidden beneath the large membrane, appearing as weak stripe in dorsal aspect, apical portion black in dorsal and lateral aspects and ending subapically (Figs 17, 19); ventral sclerite with weak ridge fringed by a row of short ventral teeth (Fig. 19). Paraproct trilobed (Figs 17–20): inner lobe slightly bilobed, relatively elongate and reach end of terminalia, basally connected to median lobe and membranous portion between the lobes bear minute apical setae; median lobe sclerotized, up-curved at apical half, apical portion in dorsal aspect with 5 or 6 medium-sized spines: 2 or 3 upper spines and 3 lateroapical spines (Figs 17, 19, 20); outer lobe sclerotized and medially curved along cerci, apex up-curved, without spines (Figs 18, 20).

Female (Fig. 15): Forewing length 7.5–7.8 mm. Sternum VII posteriorly greatly produced in a large semicircular pregenital plate, the plate mainly being pale but posterior margin brown. Sternum VIII forms bilobed subgenital plate with a deep medial indentation at inner margin of the lobes, the median notch between subgenital plate dome-like. Paragenital plate with two branches, lower plate large, rounded and lightly pigmented lobe with hairs in ventral surface, upper plate forming a dark brown sloping sclerite seemingly like the pod brim. Sternum IX with anterior margin slightly protruded medially. Paraproct and cerci brownish.

Female inner genitalia (Fig. 21): Inner structure under pregenital plate is simple and membranous, lightly sclerotized, ovum-shaped anterior shield attached to the spermathecal ductus; attached muscles linking with margin of paragenital plate easily observed.

**Figure 22.**
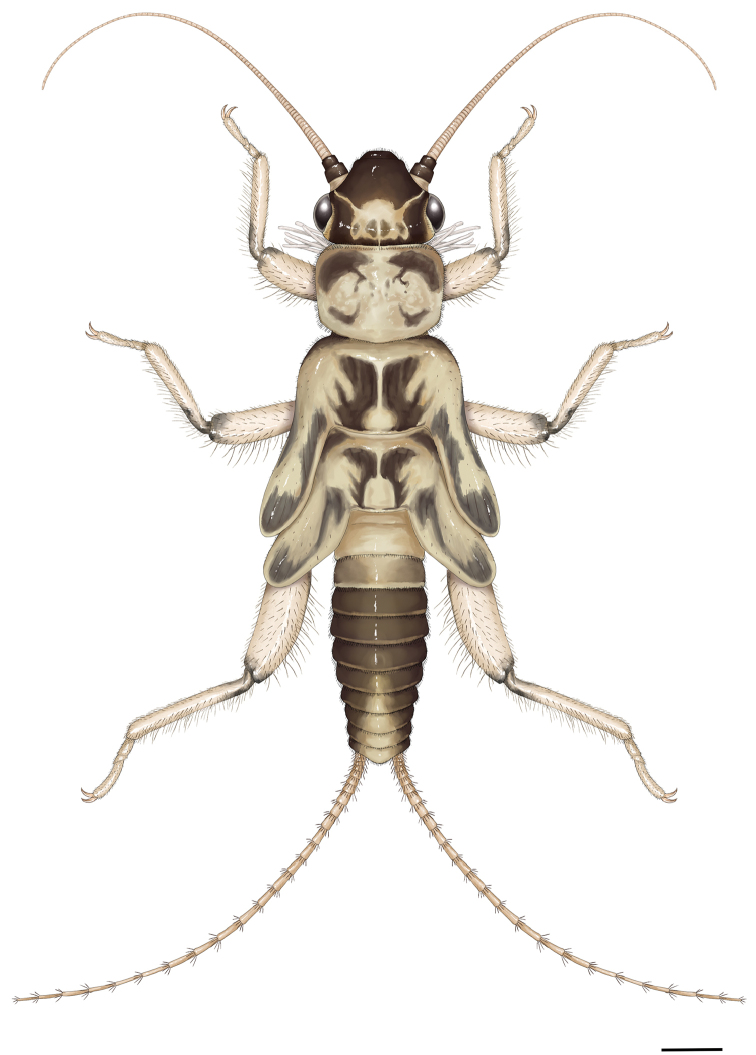
*Amphinemuradingoidea* sp. n., habitus of matured male paratype larva. Scale bar: 0.5 mm.

Mature larva (Fig. 22): Body relatively stout and small, body length without antennae and cerci 5.5 mm. General color light brown with distinct and characteristic dark brown dorsal pattern: head mostly dark brown with well delimited light brown pattern on occiput; scape and pedicel dark brown, rest of the antennae and palpi light brown; pronotum mostly pale, dark brown pattern similar to adults; meso-, metanotum and wing pads mostly pale but with distinct, paired Z-shaped dark pattern; legs light brown, apex of femora and base of tibiae darker; abdominal terga I–II entirely pale, terga III–V entire dark, terga VI–X laterally dark with medial pale area gradually widened towards the apex; cerci pale brown. Ventral aspect of the body entirely pale. Setation long and distinct. Legs moderately short, width of hind femora about one-third of their length. The pronotum is rounded trapezoidal, wider than long, slightly wider than head. Cervical gills long, inner gills with 6, outer with 7 branches. Wing pads more than twice as long as the corresponding segments. Abdomen relatively stout, integument light matt brown, first 3 abdominal segments fully, further 3 partly divided by pleura. Posterior margin of sternum IX of the male larva short and blunt triangular. Cerci long, with 25 slightly clubbed segments; length of the 15^th^ segment is more than 3 times of its width.

**Figures 23–28.**
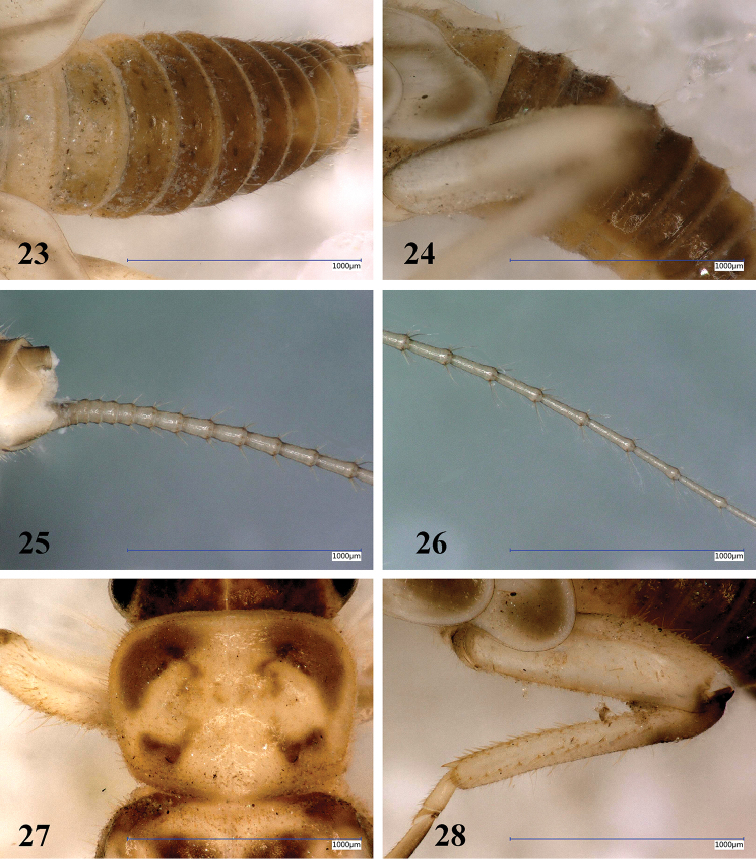
*Amphinemuradingoidea* sp. n., matured larva paratype. **23** Abdomen, dorsal view **24** Terga I–VII, lateral view **25** Paraproct and basal half of cerci, oblique ventral view **26** Cercomeres 14–21, oblique ventral view **27** Pronotum, dorsal view **28** Left hind leg, outer face. Scale bar: 1 mm.

Setation of the larva (Figs 23–28): Head, antennae and palpi with short setae. Pronotum covered with short setae; marginal setae distinct and blunt, row continuous but setae shorter in anteromedial and posteromedial third, corners have setae as long as 1/15^th^ of pronotum width (Fig. 27). Setae on meso- and metanotum with marginal setae as long as longest marginal setae on pronotum; wing pads with short setae besides marginal ones. Legs with relatively sparse but diverse setae, all tibiae bear sparse and indistinct swimming hairs as long as tibia width (Fig. 28). Longest acute setae of all femora are about as long as half of the corresponding femur width, not arranged in line but restricted to apical half. Tarsi and claws normal. Tergal segments covered with thin setae and a few short hairs; posterior margin with row of 14–16 acute and erect setae, of various length, longest reaches more than half of segment length (Figs 23, 24). Cercal segments with relatively sparse and moderately long setation, apical whorl of setae consist of both dark and whitish setae; setae sparser and shorter on the basal segments (Figs 25, 26). Cercomeres 14–16 with intercalary setae-like indistinct fine hairs, as long as the segment width, and an apical whorl of 8 or 9 acute setae that are much shorter than segment length (Fig. 26).

#### Type material.

Holotype male (HIST): CHINA, Shaanxi Province, Hanzhong, Foping County, brook in Lover’s Valley by Foping Old Town, 885 m, 33°31.838'N, 107°59.432'E, 2018.IV.21, leg. W.H. Li, R.R. Mo and D. Murányi. Paratypes: same data as holotype: 2 females, 1 male larva (HIST), 1 male, 2 females (HNHM); Shaanxi Province, Hanzhong, Foping County, Changjiaoba Town, steep forest brook in Dizhuang valley, 980 m, 33°33.543'N, 107°58.263'E, 2018.IV.21, leg. W.H. Li, R.R. Mo and D. Murányi: 3 larvae (HNHM).

#### Affinities.

On the basis of the simple epiproct and rather elongated median lobe of the paraproct, the male of *A.dingoidea* is similar to several other Chinese species, e.g. *A.curvispina* (Wu, 1973), *A.filarmia* Li & Yang, 2000, *A.microhamita* Li, Dong & Yang, 2018, and *A.ovalis* Li & Yang, 2005. However, the combination of the long and slightly bilobed inner lobe and short, spine-less outer lobe of paraproct, together with simple but apically notched epiproct, distinguish the new species from all congeners. The female can be easily distinguished from females of the hitherto known Asian *Amphinemura* species on the basis of the distinctive shape of their subgenital plate combined with rather simple inner genitalia. The larva is distinctive by its rather conspicuous pale and dark brown color pattern.

#### Distribution and ecology.

Most specimens were found along a small forest brook (Fig. 33), but a single male was collected by the upper, faster flowing section of the same large stream where *A.albicauda* was collected at the lower section (Fig. 35). Both localities are at moderate elevations, have fast current, and the littoral vegetation consists of deciduous forest, willow bush, and dense littoral grasses and sedges. The width of the brook at the type locality is less than 1 m and usually less than 10 cm in depth, but deeper, nearly stagnant pools also occur. The substrate is bedrock with small stones and variable debris (Fig. 34). Only a single mature larva was found among several other Nemouridae larvae, whereas most adults were fully mature and abundant among the adult stoneflies collected at this stream, suggesting late April is after the peak emergence. At the type locality, accompanying stoneflies were Nemouridae: *Amphinemurasinensis*, *A.unihamata* (Wu, 1973), *Sphaeronemouragrandicauda* (Wu, 1973), an unidentified *Indonemoura* sp. found only as premature larvae, and a yet undetermined *Nemoura* sp. of the *ovocercia* species group. In the Dizhuang valley, the paratype male was caught together with the recently described *Cryptoperlanangongshana* Huo & Du, 2018.

**Figures 29–30.**
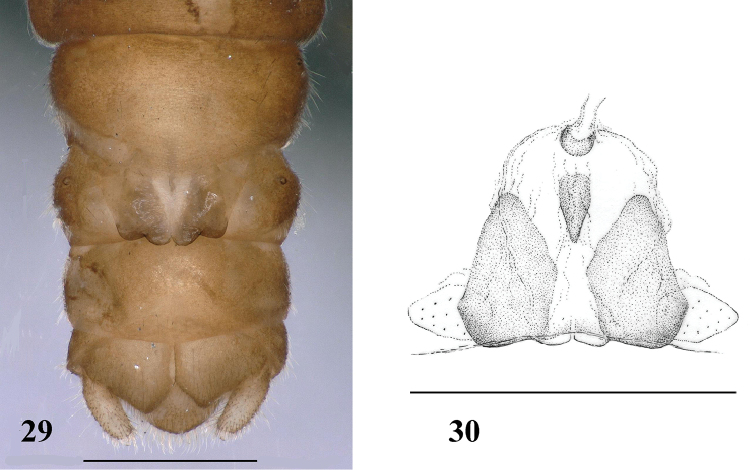
*Amphinemuraunihamata* (Wu, 1973), terminalia of adult female. **29** Terminalia in ventral view **30** Inner genitalia, dorsal view. Scale bar: 0.5 mm.

**Figures 31–34.**
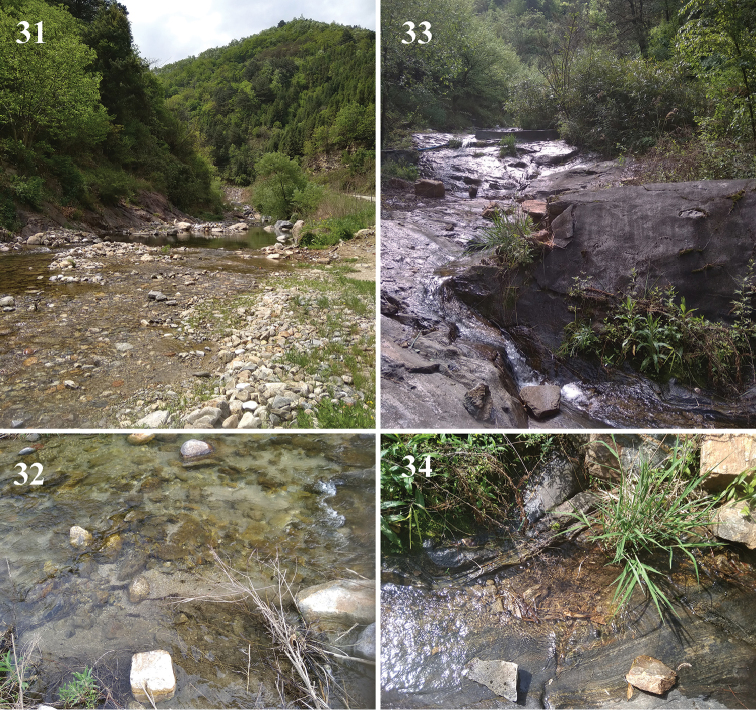
Type localities. **31, 32***Amphinemuraalbicauda* sp. n., Dizhuang valley, 895 m, 33°33.344'N, 107°59.018'E**33, 34***Amphinemuradingoidea* sp. n., Lover’s Valley, 885 m, 33°31.838'N, 107°59.432'E.

#### Etymology.

The specific name refers to the shape of the pod-like subgenital plate and the pot-like anterolateral branches, which overall resembles the Chinese “ding”, an ancient pot unique in Chinese culture.

#### Remarks.

The adults were associated with the single mature larva on the basis of the distinct pronotal pattern and similar, contrasting body color.

### Amphinemura sinensis

Taxon classificationAnimaliaPlecopteraNemouridae

(Wu, 1926)

Fig. 35

#### Material examined.

CHINA, Shaanxi Province, Hanzhong, Foping County, brook in Lover’s Valley by Foping Old Town, 870 m, 33°31.819'N, 107°59.335'E, 2018.IV.21, leg. W.H. Li, R.R. Mo and D. Murányi: 1 male (HNHM); Shaanxi Province, Hanzhong, Foping County, Changjiaoba Town, lower section of a large unnamed stream in Dizhuang valley, 895 m, 33°33.344'N, 107°59.018'E, 2018.IV.21, leg. W.H. Li, R.R. Mo and D. Murányi: 1 male (HIST).

#### Distribution and ecology.

This species was described from Jiangsu Province, later reported also from Beijing and Henan (Yang and Li 2018), and is the only member of the *sinensis* group that occurs in Palaearctic areas of China. We collected this species at the type localities of the new species above (Figs 31–34). Our new records represent the first records from Shaanxi.

**Figure 35.**
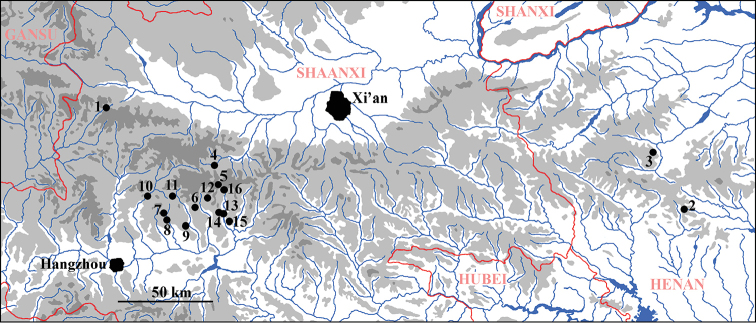
Known distribution of Amphinemurinae in the Qinling Mountains **1***Amphinemuralingulata*, *Mesonemouramembranosa***2***A.didyma*, *A.mamillata*, *Indonemouraauriformis*, *M.tritaenia***3***Sphaeronemouraseparata***4***A.annulata*, *A.lingulata***5***A.annulata***6***A.dicroidea***7***A.longihamita*, *S.grandicauda***8***A.microhamita***9***A.microhamita***10***A.microhamita*, *A.multispina***11***S.grandicauda***12***S.grandicauda***13***A.albicauda*, *A.sinensis***14***A.dingoidea***15***A.dingoidea*, *A.sinensis*, *A.unihamata*, *Indonemoura* sp., *S.grandicauda***16***A.unihamata*.

#### Remarks.

From both streams where we found this species, we also collected mature larvae that are probably belong to this species. These are very similar to the larva of *A.albicercia* in color pattern and setation, but lacks the distinctive, dense white hairs on the cerci.

### Amphinemura unihamata

Taxon classificationAnimaliaPlecopteraNemouridae

(Wu, 1973)

Figs 29 , 30 , 35

#### Description of the female.

(Fig. 29) Forewing length 9.0–9.6 mm. Sternum VII posteriorly produced in a wide but short, semicircular pregenital plate, the plate pale. Sternum VIII forms trapezoid subgenital plate that is about half as wide as the segment, posterior lobes distinctly divided into rounded sublobes, nearly equal in size; posteromedial indentation narrow and triangular, with white medial area widened towards anterior edge. Paragenital plate large, rounded, but pale and rather indistinct. Sternum IX with anterior margin slightly protruded medially. Paraproct and cerci brown.

Female inner genitalia (Fig. 30): Inner structure consist of a beak-like, dark median sclerite positioned under pregenital plate at the anterior edge of subgenital plate, is simple and a smaller but thick, ring-shaped anterior shield attached to the spermathecal ductus; membranous portion wide and attached to the paragenital plates.

#### Material examined.

CHINA, Shaanxi Province, Hanzhong, Foping County, steep forest brook in Panda valley, 1270 m, 33°40.322'N, 107°58.190'E, 2018.IV.20, leg. W.H. Li, R.R. Mo and D. Murányi: 1 male (HNHM); Shaanxi Province, Hanzhong, Foping County, brook in Lover’s Valley by Foping Old Town, 885 m, 33°31.838'N, 107°59.432'E, 2018.IV.21, leg. W.H. Li, R.R. Mo and D. Murányi: 2 females (HNHM); same brook and date, a few hundred meters downstream, 870 m, 33°31.819'N, 107°59.335'E: 1 male (HIST), 1 female (HNHM).

#### Distribution and ecology.

Previously, this species was known only from the holotype, collected in Sichuan. It was recently redescribed by Yang et al. (2015). We found it at the type locality of *A.dingoidea* (Figs 31, 32) and a similar small brook at slightly higher elevation, representing its first records from Shaanxi.

#### Remarks.

*Amphinemuraannulata* Du & Ji in Ji et al., 2014 was described one year earlier before the specific identity of *A.unihamata* was resolved by Yang et al. (2015). The holotype of *A.annulata* was designated from Zhejiang Province but the paratype series included specimens also from Shanxi, Shaanxi, and Guizhou provinces. The two species are probably conspecific, but without examination of holotype of *A.annulata*, we do not propose a synonymy of *A.unihamata* at this time.

## Concluding remarks: the Amphinemurinae known from the Qinling Mountains

Sloping from the Qinghai-Tibet Plateau to the North China Plain, the Qinling Mountains form the boundary between the Palaearctic and Oriental realms and are an important biodiversity hotspot (Chen et al. 2008, Zhang et al. 2014). Even though one of the earliest Chinese publications on stoneflies was published on species from the Qinling (Klapálek 1908), the Amphinemurinae remained unknown until the last ten years.

The first known Amphinemurinae species, *Mesonemouramembranosa* Du & Zhou in Du et al., 2007a was described from the central part of the mountain range in Shaanxi. Subsequently, four additional species were reported from the eastern extremities of the range in Henan Province: *M.tritaenia* Li & Yang, 2007b, *A.mamillata* Li & Yang, 2008e, and *Indonemouraauriformis* Li & Yang, 2008e were described as new, whereas *A.didyma* was reported from Baotianman as new for Henan (Li and Yang 2008e). Later, *Sphaeronemouraseparata* Li, Murányi & Yang, 2014 was described from the ranges in Henan, while Ji et al. (2014) described *A.annulata* Du & Ji in Ji et al., 2014 with paratypes from the Shaanxi ranges, and *A.lingulata* Du & Wang in Ji et al., 2014 from the holotype and some paratypes from the central ranges in Shaanxi. More recently, Li et al. (2018) described three species, *A.dicroidea* Li, Dong & Yang, 2018, *A.longihamita* Li, Dong & Yang, 2018, and *A.microhamita* Li, Dong & Yang, 2018, from the Shaanxi areas of the Qinling, and reported *A.multispina* (Wu, 1973) and *S.grandicauda* (Wu, 1973) from the same ranges.

Including the newly described or reported species in the present paper, 16 species are now known from the Qinling Mountains. However, this number must be just a fraction of the possible diversity. Seven of the species described from the range are known only from their Qinling type localities, whereas *A.mamillata* was later found also in Ningxia (Yang and Li 2018), and the type series of *A.annulata* included specimens from Zhejiang, Shanxi, and Guizhou. The paratypes of *A.lingulata* were from Sichuan (Ji et al. 2014). Among species subsequently reported from the Qinling, *A.didyma* is also known from Ningxia and Inner Mongolia, *A.multispina, A.unihamata*, and *S.grandicauda* from Sichuan, and *A.sinensis* from Jiangsu, Beijing, and Henan (Yang and Li 2018). These distributions support well the idea that the Qinling Mountains, as a border zone between realms, include species distributed mainly in the Palaearctic as well as other species that are members of the Oriental fauna. However, none of the known species have dispersed far into the both realms, e.g. no species is known to occur in northeastern or southern China.

*Amphinemura* is a widespread Holarctic and Oriental genus, whereas *Indonemoura* Baumann, 1975, *Mesonemoura* Baumann, 1975, and *Sphaeronemoura* Shimizu & Sivec, 2001 are distributed primarily in the Oriental Region. Among these three genera, *Indonemoura* is not known to occur on the mainland north to Qinling, further supporting the uniqueness of this Palaearctic and Oriental border region.

## Supplementary Material

XML Treatment for Amphinemura albicauda

XML Treatment for Amphinemura dingoidea

XML Treatment for Amphinemura sinensis

XML Treatment for Amphinemura unihamata

## References

[B1] Baumann RW (1975) Revision of the stonefly family Nemouridae (Plecoptera): a study of the world fauna at the generic level. Smithsonian Contributions to Zoology 211: 1–74. 10.5479/si.00810282.211

[B2] Chen L Song Y-L Xu S-F (2008) The boundary of Palaearctic and Oriental realms in western China. Progress in Natural Science 18: 833–841. 10.1016/j.pnsc.2008.02.004

[B3] DeWalt RE Maehr MD Neu-Becker U Stueber G (2018) Plecoptera Species File Online. Version 5.0/5.0. http://Plecoptera.SpeciesFile.org [Accessed on: 2018-5-6]

[B4] Du Y-Z Wang Z-J (2007) New species of the genus *Amphinemura* (Plecoptera: Nemouridae) from Yunnan, China. Zootaxa 1554: 57–62.

[B5] Du Y-Z Wang Z-J Zhou P (2007a) Two new species of the genus *Mesonemoura* and redescription of *M.spiroflagellate* (Plecoptera: Nemouridae) from China. Zootaxa 1495(1): 47–52. 10.11646/zootaxa.1495.1.3

[B6] Du Y-Z Wang Z-J Zhou P (2007b) Four new species of the genus *Amphinemura* (Plecoptera: Nemouridae) from China. Aquatic Insects 29(4): 297–305. 10.1080/01650420701552482

[B7] Huo QB Du YZ (2018) Two new species of *Cryptoperla* (Plecoptera, Peltoperlidae) from China. Zootaxa 4374(3): 395–408. 10.11646/zootaxa.4374.3.4 29689807

[B8] Ji X-Y Du Y-Z (2014) Four new species of *Amphinemura* (Plecoptera: Nemouridae) from Sichuan, China. Florida Entomologist 97(2): 692–698. 10.1653/024.097.0249

[B9] Ji X-Y Du Y-Z Wang Z-J (2014) Two new species of the stonefly genus *Amphinemura* (Insecta, Plecoptera, Nemouridae) from China. ZooKeys 404: 23–30. 10.3897/zookeys.404.7067 PMC4023258 24843269

[B10] Klapálek F (1908) Plecoptera. In: Filchner W (Ed.) Wissenschaftliche Ergebnisse der Expedition Filchner nach China und Tibet 1903–1905, 10(1), 1.Abschnitt: Zoologische Sammlungen, 2. Abschtitt: Botanische Sammlungen. Ernst Siegfried Mittler un Sohn, Berlin, 59–64. [+ pl. 4]

[B11] Klapálek F (1912) Plecoptera I – H. Sauter’s Formosa-Ausbeute. Entomologische Mitteilungen 1: 342–351. 10.5962/bhl.part.25903

[B12] Li W-H Mo R-R (2018) Two new species of *Kamimuria* (Plecoptera: Perlidae) from Shaanxi Province, China. Zootaxa 4379(4): 594–600. 10.11646/zootaxa.4379.4.11 29689970

[B13] Li W-H Murányi D (2015) A remarkable new genus of Perlodinae (Plecoptera: Perlodidae) from China, with remarks on the Asian distribution of Perlodinae and questions about its tribal concept. Zoologischer Anzeiger 259: 41–53. 10.1016/j.jcz.2015.10.003

[B14] Li W-H Yang D (2005) Two new species of *Amphinemura* (Plecoptera: Nemouridae) from Sichuan, China. Zootaxa 1083: 63–68. 10.11646/zootaxa.1083.1.3

[B15] Li W-H Yang D (2006) Three new species of *Amphinemura* (Plecoptera: Nemouridae) with a key to the species from Guizhou Province, China. Zootaxa 1154: 41–48.

[B16] Li W-H Yang D (2007a) Review of the genus *Amphinemura* (Plecoptera: Nemouridae) from Guangdong, China. Zootaxa 1511: 55–64.

[B17] Li W-H Yang D (2007b) New species of the genus *Mesonemoura* (Plecoptera: Nemouridae) from China. Aquatic Insects 29(3): 173–180. 10.1080/01650420701481518

[B18] Li W-H Yang D (2008a) Species of *Amphinemura* (Plecoptera: Nemouridae) from Tibet, China. Zootaxa 1688: 54–60. 10.11646/zootaxa.4247.4.11 28610055

[B19] Li W-H Yang D (2008b) New species of Nemouridae (Plecoptera) from China. Aquatic Insects 30(3): 205–221. 10.1080/01650420802334038

[B20] Li W-H Yang D (2008c) A new species of *Amphinemura* (Plecoptera: Nemouridae) from China. Zootaxa 1892: 65–68. 10.11646/zootaxa.5194.3.7 37045322

[B21] Li W-H Yang D (2008d) Two new species of *Amphinemura* (Plecoptera: Nemouridae) from Yunnan, China, with the redescription of *A.triramia* (Wu, 1962). Zootaxa 1926: 61–67.

[B22] Li W-H Yang D (2008e) Two new species and two new records of stonefly family Nemouridae from Henan (Plecoptera: Nemouroidea). In: Shen X Lu C (Eds) The Fauna and Taxonomy of Insects in Henan, 6.China Agricultural Science and Technology Press, Beijing, 11–16.

[B23] Li W-H Yang D (2011) Two new species of *Amphinemura* (Plecoptera: Nemouridae) from China. Zootaxa 2975: 29–34. 10.11646/zootaxa.4247.4.11 28610055

[B24] Li W-H Dong W-B Yang D (2018) New species and new records of Amphinemurinae (Plecoptera: Nemouridae) from Shaanxi Province of China. Zootaxa 4402(1): 149–162. 10.11646/zootaxa.4402.1.7 29690282

[B25] Li W-H Du K-Sh Yang D (2017a) Two new species of the nemourid genus *Amphinemura* (Plecoptera) from China. Zootaxa 4254: 485–492. 10.11646/zootaxa.4254.4.5 28609955

[B26] Li W-H Li K-F Wang R-F Yang D (2016c) The first description of the larvae of the Chinese species *Paraleuctratianmushana* Li, Yang (Plecoptera: Leuctridae). Zootaxa 4061(1): 93–100. 10.11646/zootaxa.4061.1.10 27395484

[B27] Li W-H Murányi D Pan J-J Yang D (2013) New and little known species of Nemouridae (Plecoptera) in Inner Mongolia of China. Zootaxa 3746(3): 473–480. 10.11646/zootaxa.3746.3.6 25113490

[B28] Li W-H Murányi D Yang D (2014) A new species of *Sphaeronemoura* (Plecoptera: Nemouridae) from Henan Province of China, with additions to generic characters of the female and larva. Zootaxa 3793(3): 371–378. 10.11646/zootaxa.3793.3.5 24870176

[B29] Li W-H Yang D Sivec I (2005) A new species of *Amphinemura* (Plecoptera: Nemouridae) from China. Entomological News 116(2): 93–96.

[B30] Li W-H Wang Y Yang D (2016b) Two new species of *Amphinemura* (Plecoptera: Nemouridae) from the Gaoligong Mountains of Yunnan, China. Zootaxa 4200(3): 381–388. 10.11646/zootaxa.4200.3.3 27988630

[B31] Li W-H Wang Y Yang D (2017b) Two new species of *Amphinemura* (Plecoptera: Nemouridae) from Tibet. Zootaxa 4247(4): 494–500. 10.11646/zootaxa.4247.4.11 28610055

[B32] Martynov A-B (1928) Zur Kenntnis der Plecopteren des Kaukasus. I. Nemuridae und Leuctridae des Zentralkaukasus. Travaux de la Station Biologique du Caucase du Nord 2(2–3): 18–42.

[B33] Mo R-R Yang D Wang G-Q Li W-H (2017) One new species of *Amphinemura* and description of the female of *A.ancistroidea* Li, Yang (Plecoptera: Nemouridae) from Guangxi Zhuang Autonomous Region of southern China. Zootaxa 4276(2): 277–284. 10.11646/zootaxa.4276.2.9 28610211

[B34] Ris F (1902) Die schweizerischen arten der Perliden-gattung *Nemura*. Mitteilungen der schweizerischen entomologischen Gesellschaft 10: 378–405. 10.5962/bhl.part.2751

[B35] Shimizu T (1997) Two new species of the genus *Amphinemura* from Japan and Taiwan (Plecoptera: Nemouridae). Japanese Journal of Systematic Entomology 3(1): 77–84.

[B36] Shimizu T (1998) The group of *Amphinemuramegaloba* (Plecoptera, Nemouridae). Japanese Journal of Systematic Entomology 4(2): 227–236.

[B37] Stark BP Sivec I (2010) Eight new species of *Amphinemura* (Plecoptera: Nemouridae) from Vietnam. Illiesia 6(5): 41–51.

[B38] Wang Z-J Du Y-Z Sivec I Li Z-Z (2006) Records and descriptions of some Nemouridae species (Order: Plecoptera) from Leigong Mountain, Guizhou province, China. Illiesia 2(7): 50–56.

[B39] Wang Z-J Zhang J-H Zhu J-Y (2007) A new species of the genus *Amphinemura* (Plecoptera: Nemouridae) from Xinjiang, China. Acta Zootaxonomica Sinica 29: 13–16.

[B40] Wu C-F (1926) Two new species of stoneflies from Nanking. The China Journal of Science and Arts 5(6): 331–332.

[B41] Wu C-F (1935) New species of stoneflies from East and South China. Bulletin of the Peking Society of National History 9: 227–243.

[B42] Wu C-F (1938) *Plecopterorumsinensium*: a Monograph of the Stoneflies of China (Order Plecoptera). Yenching University, Beijing, 225 pp.

[B43] Wu C-F (1949) Sixth supplement to the stoneflies of China (Order Plecoptera). Peking Natural History Bulletin 17(4): 251–256. [pls 1, 2]

[B44] Wu C-F (1962) Results of the Zoologico-Botanical Expedition to Southwest China, 1955–1957 (Plecoptera). Acta Entomologica Sinica 11(suppl.): 139–153.

[B45] Wu C-F (1973) New species of Chinese stoneflies (Order Plecoptera). Acta Entomologica Sinica 16(2): 111–118.

[B46] Yang D Li W-H (2018) Species Catalogue of China, Volume 2 Animals, Insecta (III), Plecoptera. Science Press Beijing, 71 pp.

[B47] Yang D Li W-H Zhu F (2004) A new species of *Amphinemura* (Plecoptera: Nemouridae) from China. Entomological News 115(4): 226–228.

[B48] Yang D Li W-H Zhu F (2015) Fauna Sinica, Insecta Vol. 58, Plecoptera: Nemouroidea. Science Press, Beijing, 518 pp.

[B49] Yang D Li W-H Sivec I (2005) A new species of *Amphinemura* from south China (Plecoptera: Nemouridae). Zootaxa 805: 1–4.

[B50] Zhang Y-B Guo L-L Wang W Li J-S (2014) Spatial distribution patterns of species richness and hotspots of protected plants in Qinling Mountain. Acta Ecologica Sinica 34(8): 2109–2117. 10.5846/stxb201311082697

[B51] Zhu F Yang D (2002) Three new species of *Amphinemura* from China (Plecoptera: Nemouridae). Acta Zootaxonomica Sinica 27(4): 745–749.

[B52] Zhu F Yang D (2003) Three new species of *Amphinemura* (Plecoptera: Nemouridae) from Tibet, China. Entomologia Sinica 10(1): 51–56.

[B53] Zwick P (2010) New species and new records of Plecoptera from Korea and the Russian Far East. Illiesia 6(9): 75–97.

